# Kampo formulas alleviate aging-related emotional disturbances and neuroinflammation in male senescence-accelerated mouse prone 8 mice

**DOI:** 10.18632/aging.203811

**Published:** 2022-01-03

**Authors:** Naoki Ito, Akiko Maruko, Kenshiro Oshima, Masaaki Yoshida, Kengo Honma, Chika Sugiyama, Takayuki Nagai, Yoshinori Kobayashi, Hiroshi Odaguchi, Norihiro Okada

**Affiliations:** 1 Oriental Medicine Research Center, Kitasato University, Tokyo 108-8642, Japan; 2 Laboratory of Genomics for Health and Longevity, School of Pharmacy, Kitasato University, Sagamihara, Kanagawa 252-0373, Japan; 3 Research Laboratory, Kotaro Pharmaceutical Co., Ltd., Hakusan, Ishikawa 920-0201, Japan; 4 Graduate School of Infection Control Sciences, Kitasato University, Tokyo 108-8642, Japan; 5 Graduate School of Medical Sciences, Kitasato University, Sagamihara, Kanagawa 252-0373, Japan; 6 Laboratory of Biochemical Pharmacology for Phytomedicines, Ōmura Satoshi Memorial Institute, Kitasato University, Tokyo 108-8642, Japan; 7 Department of Pharmacognosy, School of Pharmacy, Kitasato University, Minato-ku, Tokyo 108-8642, Japan

**Keywords:** aging, emotional disturbances, kososan, neuroinflammation, SAMP8 mice

## Abstract

Aging-induced neuroinflammation, also known as neuroinflammaging, plays a pivotal role in emotional disturbances, including depression and anxiety, in older individuals, thereby leading to cognitive dysfunction. Although numerous studies have focused on therapeutic strategies for cognitive impairment in older individuals, little research has been performed on treating its preceding emotional disturbances. Here, we examined whether Kampo formulas (kososan [KS], nobiletin-rich kososan [NKS], and hachimijiogan [HJG]) can ameliorate aging-induced emotional disturbances and neuroinflammation in mice. The depression-like behaviors observed in SAMP8 mice, relative to normally aging SAMR1 mice, were significantly prevented by treatment with Kampo formulas for 13 weeks. Western blot analysis revealed that hippocampal neuroinflammation was significantly abrogated by Kampo formulas. KS and NKS also significantly attenuated the hippocampal neuroinflammatory priming induced by lipopolysaccharide (LPS, 0.33 mg/kg, *i.p.*) challenge in SAMP8 mice. Hippocampal IL-1β, IL-6, and MCP-1 levels were significantly decreased in NKS-treated SAMP8 mice. KS and NKS showed significantly reduced tau accumulation in the brains of SAMP8 mice. RNA-sequencing revealed that each Kampo formula led to unique dynamics of hippocampal gene expression and appeared to abrogate hippocampal inflammatory responses. HJG significantly blocked the LPS-induced increase in serum IL-6 and MCP-1. These results suggest that Kampo formulas would be useful for treating aging-induced depression, in part by regulating neuroinflammatory pathways. This finding may pave the way for the development of therapeutic strategies for aging-related emotional disturbances, which may contribute to the prevention of cognitive dysfunction in older individuals.

## INTRODUCTION

Aging is a risk factor for various diseases, including emotional disorders [1, 2], neurodegenerative diseases [3], anorexia [4], diabetes [5], cardiovascular diseases [6], osteoporosis [7], presbyopia [8], hypoacusis [9], and sarcopenia [10]. Most of these diseases are linked to chronic inflammation associated with aging [11]. Numerous studies have demonstrated that neurodegenerative diseases are strongly associated with neuroinflammation, which is aggravated by aging (termed neuroinflammaging [12]) [13–15].

Recent studies have also reported that neuroinflammation plays an important role in the pathology of emotional disorders, including depression [16–20]. This suggests a close link between neuroinflammaging and depression, as demonstrated by our recent finding that neuroinflammaging underlies emotional disturbances in a senescence-accelerated mouse model [21]. Several animal and human studies have reported that existing antidepressants attenuate neuroinflammation [22–25], but because antidepressants affect the metabolism of glucose [26], lipids [27], and other drugs [28–31] and may lead to exacerbation of diseases comorbid with depression, attention should be paid to long-term antidepressant treatment in older individuals with polypharmacy. Moreover, a recent finding that showed that depression causes cognitive dysfunction deterioration in older individuals with small vessel disease [32] suggests that the onset of cognitive impairment could be accelerated by depressive pathology [33]. These findings underscore the need for treating depression in older individuals. It is thus desirable to establish safer and more-effective therapeutic strategies and develop alternative therapies for late-life depression.

Although antidepressants are available as general medication for depression, some Kampo formulas, which are traditional Japanese herbal medicines, have clinically beneficial effects on emotional disorders, including depressive mood and anxiety, in patients with widely varying age ranges. Among these, kososan (KS), which consists of five herbs (Cyperus rhizome, Perilla herb, *Citrus unshiu* peel, Glycyrrhiza, and Ginger), is currently used to treat depressive states as well as the common cold, allergic urticaria caused by ingested food, irritable bowel syndrome, chronic fatigue syndrome, insomnia, and autonomic imbalance. Our previous animal studies using experimental stress models have demonstrated that KS exerts antidepressant-like effects by normalizing the dysfunction of the hypothalamic–pituitary–adrenal axis [34, 35], regulating the orexin A/neuropeptide Y signaling system [36, 37], and modulating metabotropic glutamate receptor 2 and 2’,3’-cyclic nucleotide 3’-phosphodiesterase 1 in the hypothalamus, according to a proteomic analysis [38]. Our recent study also found that KS prevented social avoidant behavior, a depressive-like behavior, presumably by attenuating hippocampal neuroinflammation in a social-defeat mouse model of depression [39]. Accumulating evidence has revealed that nobiletin, a polymethoxyflavone present in *Citrus unshiu* peel, has anti-inflammatory [40, 41], neuroprotective [42], and antidepressant-like activities [40, 43–45] and improves cognitive impairment [41, 46, 47] in rodent studies. In addition, *Citrus reticulata* peel, which has a nobiletin content that is ~16-fold higher than that of *Citrus unshiu* peel, facilitates cAMP/PKA/ERK/CREB signaling in cultured hippocampal neurons [48] and may prevent the progression of cognitive dysfunction in patients with Alzheimer’s disease [49]. Although no evidence has been reported on the effects of *Citrus reticulata* peel on emotional disturbances and neuroinflammation, it would be predicted that KS containing *Citrus reticulata* peel (nobiletin-rich KS [NKS]) is more effective for treating aging-induced emotional disturbances and neuroinflammation. Given these findings and assumptions, we hypothesized that KS would ameliorate depressive-like behaviors and neuroinflammation associated with aging and that the effects of NKS would be superior to those of KS.

To evaluate this hypothesis, we examined whether KS and NKS exerted antidepressant-like effects and neuroinflammation-suppressive effects in senescence-accelerated mouse prone 8 (SAMP8) mice, which show depressive- and anxiety-like behaviors, circadian rhythm disruption, and neuroinflammation associated with aging [21]. Furthermore, we compared the effects of KS and NKS with those of another Kampo formula, hachimijiogan (HJG), which has traditionally been used to treat several aging-related disorders, including cognitive impairments [50, 51] and irritable bladder [52].

## RESULTS

### Kampo formulas ameliorate aging-induced depression-like behaviors in SAMP8 mice

**Figure 1:**
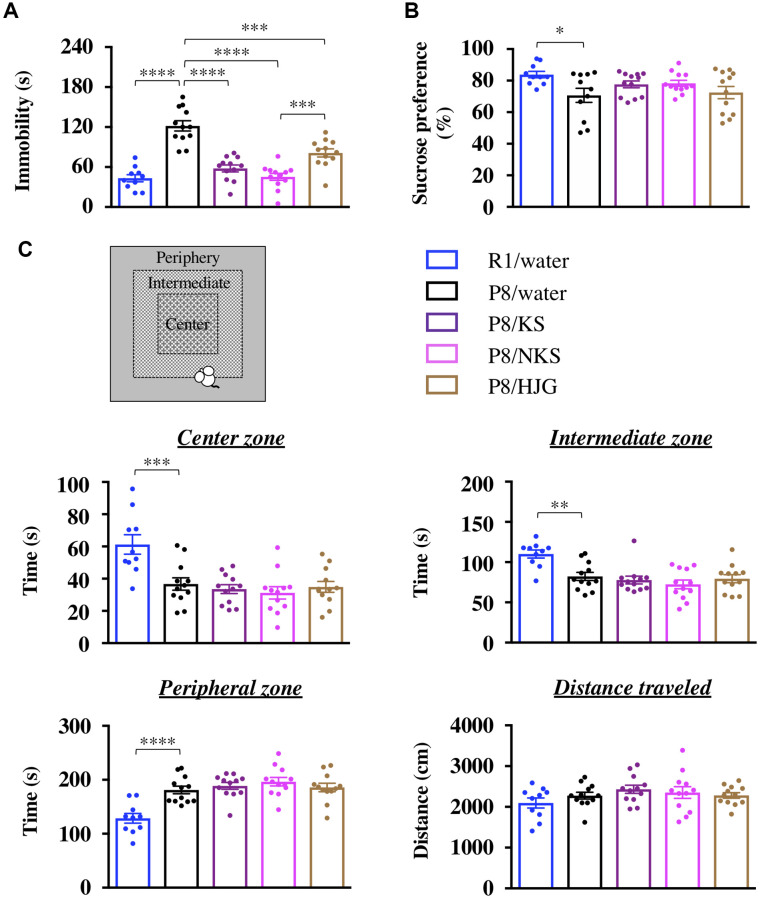
**Oral administration of Kampo formulas ameliorates depression-like behaviors but not anxiety-like behaviors in SAMP8 mice.** The tail suspension test (TST, **A**), sucrose preference test (SPT, **B**), and open field test (OFT, **C**) were performed following treatment with the indicated Kampo formulas or with water for 13 weeks. Data are shown as the mean ± SEM (*n* = 10−12). ^*^P < 0.05, ^**^P < 0.01, ^***^P < 0.001, and ^****^P < 0.0001 using Bonferroni’s post-hoc test. Abbreviations: KS: kososan; NKS: nobiletin-rich kososan; HJG: hachimijiogan.

In the TST, the immobility time of SAMP8 mice was significantly increased relative to that of senescence-accelerated mouse resistant 1 (SAMR1) mice, as normal aging controls (Figure 1A; Bonferroni’s post-hoc test, *P* < 0.0001). This effect was significantly prevented by treatment with Kampo formulas (Bonferroni’s post-hoc test; KS, *P* < 0.0001; NKS, *P* < 0.0001; HJG, *P* < 0.001). Moreover, a significant difference in immobility was found between NKS and HJG treatment (Bonferroni’s post-hoc test, *P* < 0.001), suggesting that the preventive effect of NKS was superior to that of HJG.

In the SPT, the sucrose preference was significantly lower in SAMP8 mice than in SAMR1 mice (Figure 1B; Bonferroni’s post-hoc test, *P* < 0.05). This decrease in sucrose preference was not affected by treatment with Kampo formulas. However, a significant negative correlation was also found between the immobility time in the TST and the sucrose preference in the SPT (Supplementary Figure 1; Pearson’s correlation coefficient test, *r* = −0.3048, *P* < 0.05), suggesting that Kampo formulas ameliorate aging-induced apathy-like behavior, along with anhedonia-like behavior, in SAMP8 mice. This behavioral recovery indicates the antidepressant-like effect of the Kampo formulas.

In the OFT, the time spent in the center and intermediate zones was significantly decreased and the time spent in the peripheral zone was significantly increased in SAMP8 mice relative to SAMR1 mice (Figure 1C; Bonferroni’s post-hoc test; center zone, *P* < 0.001; intermediate zone, *P* < 0.01; peripheral zone, *P* < 0.0001). These changes were retained after treatment with Kampo formulas. There was also no difference among the groups in the distance traveled during the OFT. Thus the Kampo formulas do not prevent aging-induced anxiety-like behaviors in SAMP8 mice.

**Figure 2:**
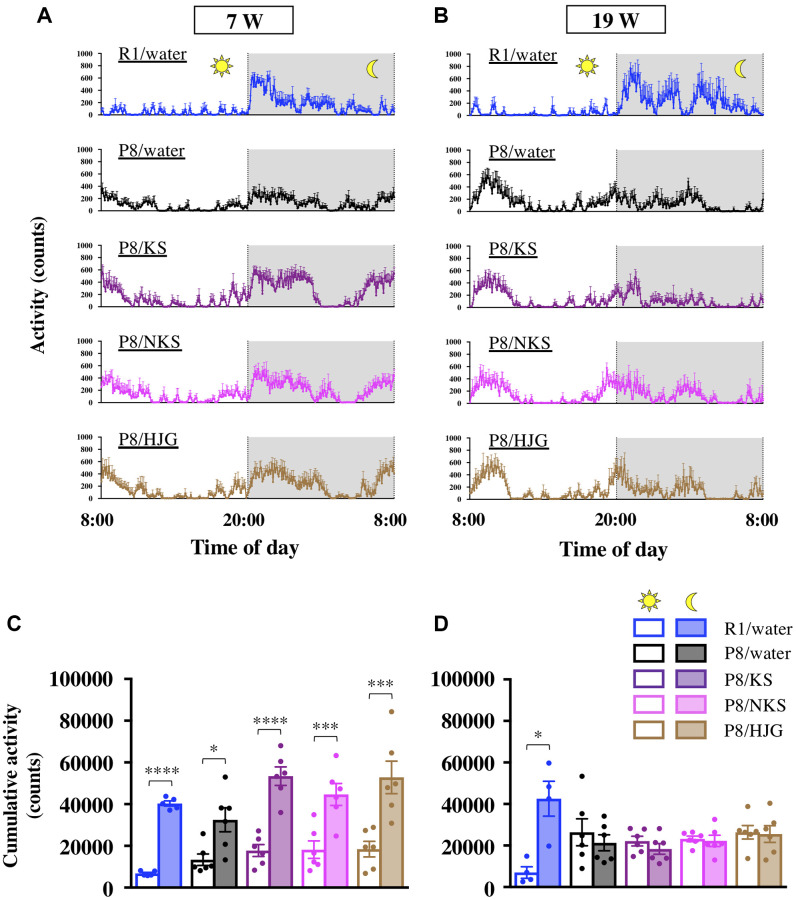
**Oral administration of Kampo formulas does not improve circadian rhythm disruption in SAMP8 mice.** Time course of locomotor activity of mice aged 7 weeks (**A**) and 19 weeks (**B**) in their home cages. The time between 8:00 and 20:00 and between 20:00 and 8:00 represents the diurnal and nocturnal phases, respectively. The cumulative activity during the diurnal and nocturnal phases of mice aged 7 weeks (**C**) and 19 weeks (**D**) is shown. Data are shown as the mean ± SEM (*n* = 4−6). ^*^P < 0.05, ^**^P < 0.01, ^***^P < 0.001, and ^****^P < 0.0001 using paired *t*-test. Abbreviations: KS: kososan; NKS: nobiletin-rich kososan; HJG: hachimijiogan; W: weeks of age.

At the beginning of the Kampo treatment period (7 weeks of age), mice in all groups had increased locomotor activity during the nocturnal phase relative to the diurnal phase (Figure 2A). Similarly, cumulative activity during the nocturnal phase was significantly higher than that during the diurnal phase for all groups (Figure 2C; paired *t*-test; SAMR1/water, *P* < 0.0001; SAMP8/water, *P* < 0.05; SAMP8/KS, *P* < 0.0001; SAMP8/NKS, *P* < 0.001; SAMP8/HJG, *P* < 0.001), indicating that both SAMR1 and SAMP8 mice showed normal circadian rhythms at 7 weeks of age. At 19 weeks of age, however, there was no difference in cumulative activity between diurnal and nocturnal phases in the water-administered SAMP8 mice (Figure 2B, 2D). There was still a significant difference between these phases in the water-administered SAMR1 mice (paired *t*-test, *P* < 0.05), although a nanotag datum of a water-administered SAMR1 mouse was not extracted because of the built-in battery exhaustion of nanotag. These results suggest that SAMP8 mice aged 19 weeks exhibit circadian rhythm disruption. This disruption was not alleviated by treatment with Kampo formulas, even when the SAMP8 mice were assessed at earlier ages (Supplementary Figure 2), implying that Kampo formulas are ineffective for treating circadian rhythm disruption in SAMP8 mice.

With regard to body weight changes, the water-administered SAMP8 mice had significantly greater body weight gain from 7 to 19 weeks of age as compared with the water-administered SAMR1 mice (Bonferroni’s post-hoc test, *P* < 0.05). No differences in body weight were found between water-administered and Kampo formulas–administered SAMP8 mice (Supplementary Figure 3).

### Hippocampal neuroinflammatory priming is attenuated in different ways by Kampo formulas in SAMP8 mice

**Figure 3:**
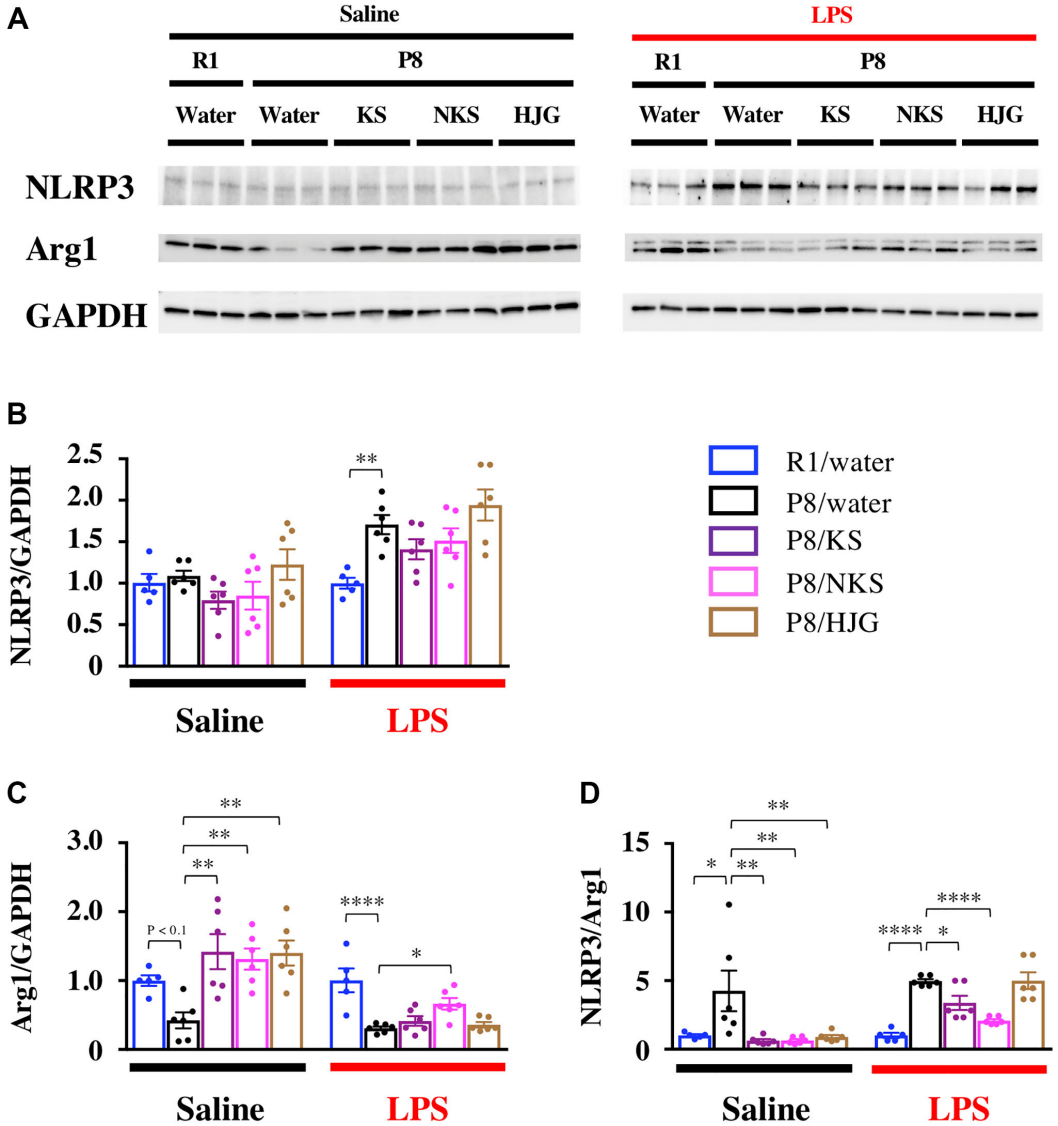
**Distinct efficacy of different Kampo formulas for blocking neuroinflammatory priming in the hippocampus of SAMP8 mice at 19 weeks of age.** (**A**) Representative western blotting images of NLRP3, Arg1, and GAPDH expression. Expression of NLRP3 (**B**) and Arg1 (**C**) were normalized based on GAPDH expression. (**D**) Pro-inflammatory/anti-inflammatory balance. Data are shown as the mean ± SEM (*n* = 5 or 6). ^*^P < 0.05, ^**^P < 0.01, ^***^P < 0.001, and ^****^P < 0.0001 using Bonferroni’s post-hoc test. Abbreviations: KS: kososan; NKS: nobiletin-rich kososan; HJG: hachimijiogan; LPS: lipopolysaccharide.

To unravel the mechanisms underlying the antidepressant-like effect of Kampo formulas, we examined whether Kampo formulas regulate neuroinflammation in the hippocampus, which is involved in the depressive phenotype [53–55]. We used expression of NLRP3 and Arg1 as markers of pro- and anti-inflammatory phenotypes, respectively. Under physiological conditions, there was no difference in NLRP3 expression among the groups (Figure 3A and 3B, left), whereas Arg1 expression tended to decrease in the water-administered SAMP8 mice relative to the water-administered SAMR1 mice (Figure 3A and 3C, left; Bonferroni’s post-hoc test, *P* < 0.1). The decrease in Arg1 expression was significantly increased by treatment with Kampo formulas (Bonferroni’s post-hoc test, *P* < 0.01). The ratio of NLRP3 to Arg1, which represents the balance between pro- and anti-inflammatory phenotypes [21], was significantly increased in the water-administered SAMP8 mice as compared with the water-administered SAMR1 mice (Figure 3D, left; Bonferroni’s post-hoc test, *P* < 0.05). This increase was significantly reduced in SAMP8 mice treated with Kampo formulas (Bonferroni’s post-hoc test, *P* < 0.01), suggesting that Kampo formulas abrogate hippocampal neuroinflammation in SAMP8 mice aged 19 weeks.

Next, we examined whether Kampo formulas prevented the neuroinflammatory response to LPS injection, to assess the influence of neuroinflammatory priming on the hippocampus. NLRP3 expression was significantly increased in the water-administered SAMP8 mice as compared with the water-administered SAMR1 mice (Figure 3A and 3B, right; Bonferroni’s post-hoc test, *P* < 0.01), but this increase was not decreased by treatment with Kampo formulas. In contrast, Arg1 expression was significantly reduced in the water-administered SAMP8 mice as compared with the water-administered SAMR1 mice (Figure 3A and 3C, right; Bonferroni’s post-hoc test, *P* < 0.0001), and the Arg1 expression was significantly increased in the NKS-administered SAMP8 mice relative to the water-administered SAMP8 mice (Bonferroni’s post-hoc test, *P* < 0.05). The ratio of NLRP3 to Arg1 was significantly increased in the water-administered SAMP8 mice as compared with the water-administered SAMR1 mice (Figure 3D, right; Bonferroni’s post-hoc test, *P* < 0.0001). This increase in the NLRP3/Arg1 ratio was significantly decreased in KS- and NKS-administered SAMP8 mice (Bonferroni’s post-hoc test; KS, *P* < 0.05; NKS, *P* < 0.0001). These results suggest that hippocampal neuroinflammatory priming by LPS is buffered by treatment with KS and NKS.

The expression of Toll-like receptor 4 (TLR4), an endogenous receptor for LPS, was also unaltered in all groups, irrespective of whether LPS had been administered (Supplementary Figure 4). This suggests that the inhibitory effects of KS and NKS on hippocampal neuroinflammatory priming do not result from the downregulation of TLR4 protein.

### Kampo formulas result in characteristic gene expression patterns in the hippocampus of SAMP8 mice

**Figure 4:**
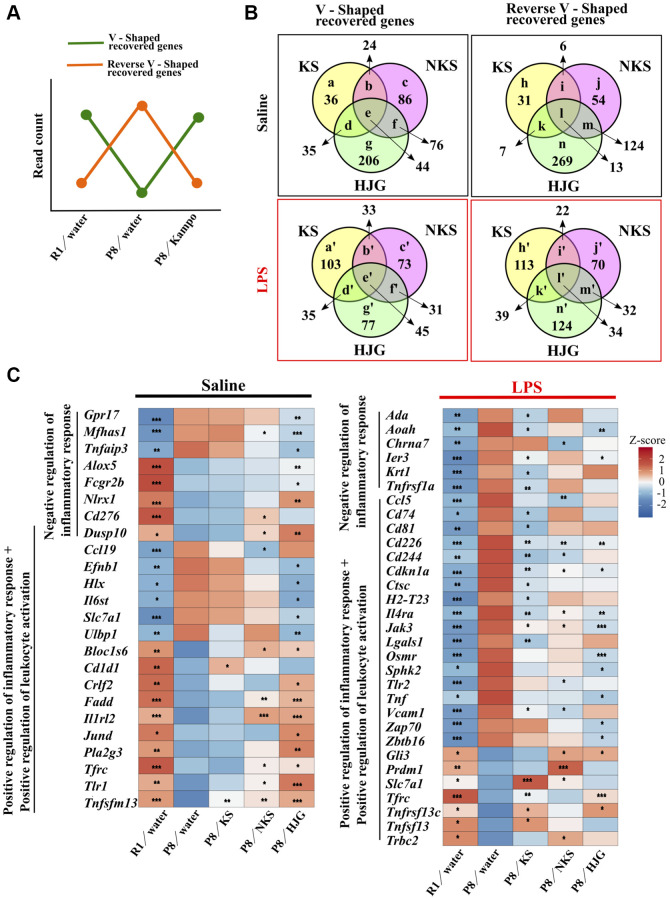
**Oral administration of Kampo formulas results in characteristic gene expression patterns in the hippocampus of 19-week-old SAMP8 mice.** (**A**) Genes that were significantly down-regulated in the water-administered SAMP8 mice as compared with water-administered SAMR1 mice and that were significantly up-regulated by treatment with Kampo formulas, i.e., the green line, are referred to as V-shaped recovered genes. Genes that were significantly up-regulated in the water-administered SAMP8 mice as compared with water-administered SAMR1 mice and that were significantly down-regulated by treatment with Kampo formulas, i.e., the orange line, are referred to as reverse V-shaped recovered genes. (**B**) Venn diagrams showing differences and similarities in gene expression among Kampo formula–treated groups. Numbers in circles indicate the number of genes with statistically significant changes in expression (*P* < 0.05). The full list of genes depicted using lower-case letters is shown in Supplementary Table 1. (**C**) Heatmap of differential gene expression related to the inflammatory response in the hippocampus of SAMR1 and SAMP8 mice. ^*^P < 0.05, ^**^P < 0.01, and ^***^P < 0.001 (*n* = 5) vs. water-administered SAMP8 mice, using the likelihood ratio test. Abbreviations: KS: kososan; NKS: nobiletin-rich kososan; HJG: hachimijiogan; LPS: lipopolysaccharide.

To investigate whether Kampo formulas have characteristic effects on hippocampal gene expression dynamics, a genome-wide RNA-seq analysis was conducted on hippocampal tissue from the treated mice. Genes that were significantly downregulated in the water-administered SAMP8 mice as compared with the water-administered SAMR1 mice and that were significantly upregulated by treatment with Kampo formulas were regarded as “V-shaped recovered genes.” Genes that were significantly upregulated in the water-administered SAMP8 mice as compared with the water- administered SAMR1 mice and that were significantly downregulated by treatment with Kampo formulas were regarded as “reverse V-shaped recovered genes.” On the basis of these criteria (Figure 4A), the V-shaped and reverse V-shaped recovered genes under physiological and LPS-stimulated conditions are summarized (Figure 4B), and a full list of significantly regulated genes is shown in Supplementary Table 1. The Venn diagrams show that there were many genes that were significantly recovered by each Kampo formula. Among the Kampo formulas, the greatest number of genes, with or without annotation, was altered by HJG treatment (Supplementary Figure 5).

To explore the biological significance of these recovered DEGs, we used the DAVID functional annotation tool. GO analysis of the genes recovered by each Kampo formula was performed, and the top 25 statistically significant (likelihood ratio test, *P* < 0.05) terms are shown in Supplementary Figure 6. The group of genes that were recovered by KS treatment showed almost no significantly enriched GO and KEGG terms. In contrast, the group of genes that were recovered after treatment with HJG had the most enriched GO and KEGG terms. Among the V-shaped recovered genes, under physiological conditions, the most enriched GO term was chemotaxis, for both NKS and HJG treatment (Supplementary Figure 6A). In terms of the reverse V-shaped recovered genes, the most-enriched GO terms were related to the regulation of transcription (Supplementary Figure 6B). In contrast, under LPS-stimulated conditions, the V-shaped recovered genes showed that the most enriched GO term was bone mineralization in KS treatment and cell cycle in HJG treatment, while few enriched GO terms were shown in NKS treatment (Supplementary Figure 6C). In terms of the reverse V-shaped recovered genes, the most-enriched GO terms for all KS, NKS and HJG treatment were related to the immune system (Supplementary Figure 6D). Several terms related to response to virus and interferon response, as well as other immune-related terms, were significantly enriched by HJG treatment.

A KEGG pathway analysis revealed that signaling pathways related to various cellular functions (e.g., cell survival, proliferation, apoptosis, cancer) were more enriched in both V-shaped (Supplementary Figure 6E and 6G) and reverse V-shaped recovered genes (Supplementary Figure 6F and 6H) associated with HJG treatment. Additionally, in the reverse V-shaped recovered genes, virus-related terms were also observed under LPS stimulation in the groups that received KS or HJG treatment (Supplementary Figure 6H). These results indicate that LPS-induced immune, inflammatory, and viral responses in the hippocampus were downregulated by treatment with Kampo formulas, and with HJG in particular.

Next, we focused on the recovered transcripts classified into the GO category “positive and negative regulation of inflammatory response” and “positive regulation of leukocyte activation.” Under physiological conditions, significant differences in numerous genes related to the inflammatory response were found between water-administered SAMR1 and water-administered SAMP8 mice, and these were significantly regulated by treatment with Kampo formulas, particularly HJG (Figure 4C, left). Similarly, under LPS-stimulated conditions, significant differences in numerous genes related to the inflammatory response were found between water-administered SAMR1 and water-administered SAMP8 mice, but there were a number of genes that were significantly recovered by treatment with KS, NKS, and HJG (Figure 4C, right). These results imply that each Kampo formula induces a characteristic gene expression pattern in the hippocampus of SAMP8 mice.

### Hippocampal tau levels are suppressed by KS and NKS treatment in SAMP8 mice

**Figure 5:**
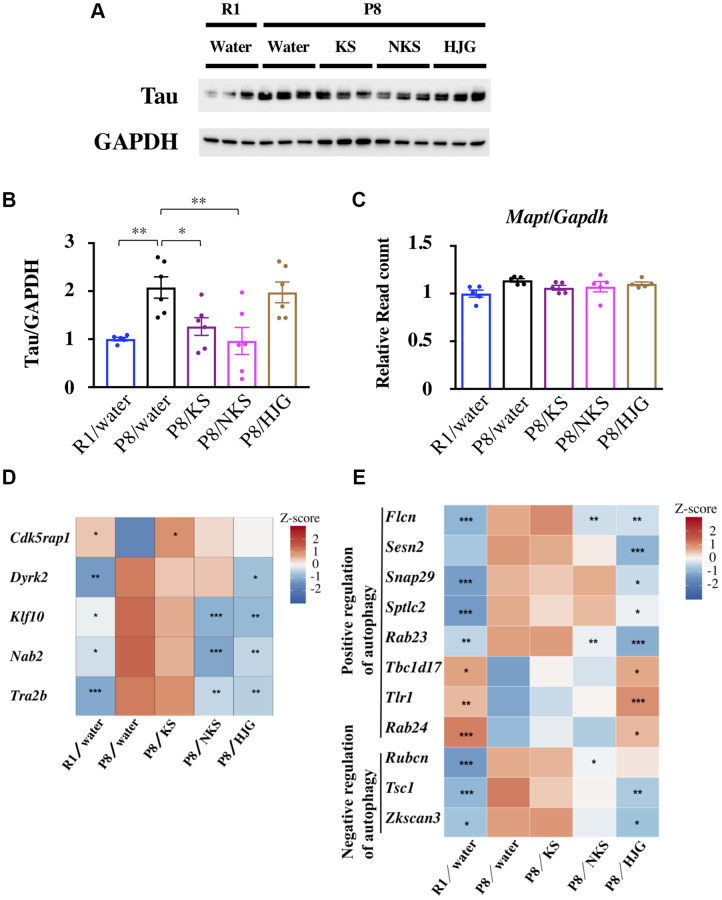
**Oral administration of KS and NKS blocks the increase in the level of tau observed in the hippocampus of SAMP8 mice.** (**A**) Representative western blot images of tau and GAPDH expression. (**B**) Expression of tau was normalized based on GAPDH expression. (**C**) Expression of *Mapt* was normalized based on *Gapdh* expression. Heatmap of differential gene expression related to phosphorylation and splicing of tau (**D**) and autophagy (**E**) in the hippocampus of mice. Data are shown as the mean ± SEM (*n* = 5 or 6). ^*^P < 0.05, ^**^P < 0.01, and ^***^P < 0.001 using Bonferroni’s post-hoc test. Abbreviations: KS: kososan; NKS: nobiletin-rich kososan; HJG: hachimijiogan.

Depression with mild cognitive impairment is associated with progression to Alzheimer’s disease [56]. Consequently, we examined the influence of Kampo formulas on tau levels in the hippocampus of 19-week-old SAMP8 mice. Hippocampal tau levels were significantly higher in the water-administered SAMP8 mice than in the water-administered SAMR1 mice (Figure 5A and 5B, Bonferroni’s post-hoc test, *P* < 0.01). These levels were significantly reduced by treatment with KS and NKS (Bonferroni’s post-hoc test; KS, *P* < 0.05; NKS, *P* < 0.01). However, no change was found between groups in terms of tau synthesis *per se*, as indicated by the gene expression levels of *Mapt*, the gene encoding tau (Figure 5C). These results suggest that tau accumulation, but not its synthesis, is hindered by treatment with KS and NKS. This finding is consistent with observations made in coronal brain sections (Supplementary Figure 7), suggesting that KS and NKS exert a suppressive effect on tau accumulation in the brain.

Next, to identify the potential sites of action of Kampo formulas for abrogating tau accumulation, we assessed the expression of genes involved in tau phosphorylation (Figure 5D). *Cdkrap1*, a gene encoding an inhibitor of cyclin-dependent protein kinase 5 (Cdk5) [57] that phosphorylates tau, showed V-shaped recovered expression with KS treatment. The gene encoding dual-specificity tyrosine phosphorylated and regulated kinase 2 (*Dyrk2*) [58] showed reverse V-shaped recovered expression with HJG treatment. NKS and HJG treatment both caused reverse V-shaped recovered of the genes encoding Krüppel-like factor-10 (*Klf10*) and Ngfi-A-binding protein-2 (*Nab2*), which mediate neurotoxicity and tau phosphorylation, respectively [59]. Furthermore, expression of the gene encoding TRA2B (*Tra2b*), a splicing factor that contributes to alternative splicing of tau exon 10 in the central nervous system, was also reversed with NKS and HJG treatment. The alternative usage of exon 10 results in tau isoforms containing either three or four microtubule-binding repeats (3R-tau and 4R-tau, respectively) [60]. Thus, changes in *Tra2b* expression in SAMP8 mice may affect the 3R-tau/4R-tau expression ratio.

Autophagy is a degradation pathway involved in the clearance of various toxic aggregated proteins associated with neurodegenerative diseases, such as tau in tauopathies [61]. Therefore, we searched for genes related to autophagy regulation among the recovered genes. As shown in Figure 5E, several autophagy-related genes were detected among both V-shaped and reverse V-shaped recovered genes associated with NKS and HJG treatment. These results suggest that reduction of tau protein caused by KS and NKS treatment is more likely due to inhibition of tau phosphorylation than to tau degradation by autophagy. However, it remains uncertain why HJG failed to reduce tau levels, regardless of marked changes in genes related to tau dynamics.

### Hippocampal levels of pro- and anti-inflammatory cytokines and chemokines are modulated by NKS treatment

**Figure 6:**
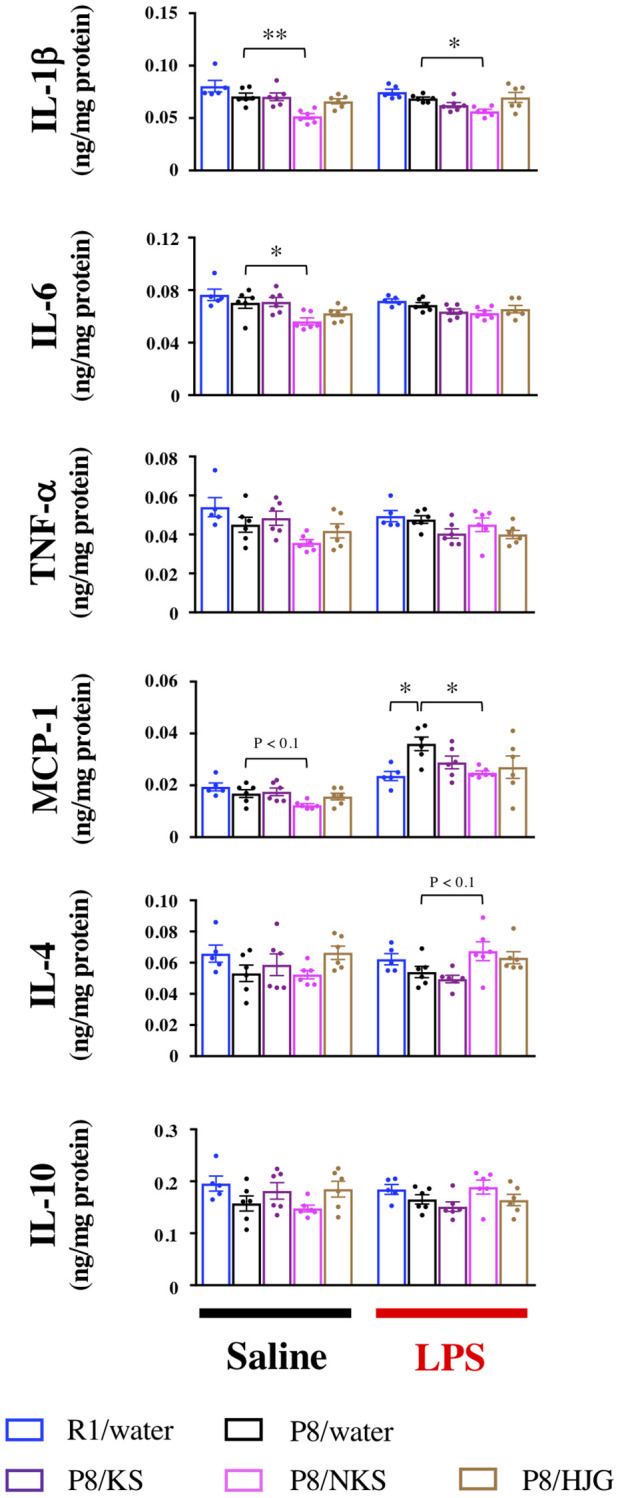
**Oral administration of NKS modulates pro- and anti-inflammatory cytokine and chemokine levels in the hippocampus of mice after saline or LPS injection.** Data are shown as the mean ± SEM (*n* = 5 or 6). ^*^P < 0.05 and ^**^P < 0.01 using Bonferroni’s post-hoc test. Abbreviations: KS: kososan; NKS: nobiletin-rich kososan; HJG: hachimijiogan; LPS: lipopolysaccharide.

To verify the effects of Kampo formulas on hippocampal neuroinflammation in western blotting analysis, we measured hippocampal protein levels of pro- and anti-inflammatory cytokines and chemokines by ELISA. Under physiological conditions, IL-1β and IL-6 levels were significantly reduced in the NKS-administered SAMP8 mice as compared with the water-administered SAMP8 mice (Figure 6; Bonferroni’s post-hoc test; IL-1β, *P* < 0.01; IL-6, *P* < 0.05). The NKS-administered SAMP8 mice also showed a tendency for reduced MCP-1 levels as compared with the water-administered SAMP8 mice (Bonferroni’s post-hoc test, *P* < 0.1). Under LPS-stimulated conditions, IL-1β and MCP-1 levels were significantly reduced in the NKS-administered SAMP8 mice as compared with the water-administered SAMP8 mice (Bonferroni’s post-hoc test, IL-1β, *P* < 0.05; MCP-1, *P* < 0.05). The NKS-administered SAMP8 mice also showed a trend toward elevated IL-4 levels as compared with the water-administered SAMP8 mice (Bonferroni’s post-hoc test, *P* < 0.1). These results partially reflected data obtained from western blotting analysis, particularly in the NKS-treated group, which suggested that NKS plays a role in preventing hippocampal neuroinflammation and neuroinflammatory priming, thereby leading to antidepressant-like effects.

### Oral administration of HJG suppresses systemic inflammation in SAMP8 mice

**Figure 7:**
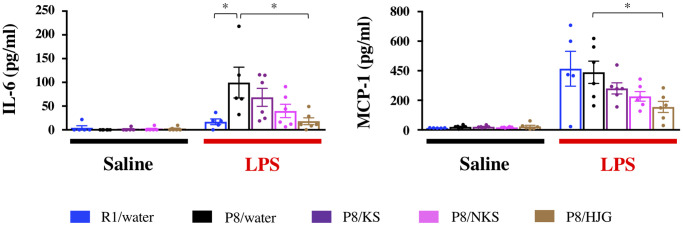
**Serum levels of a pro-inflammatory cytokine (IL-6) and chemokine (MCP-1) are suppressed by oral administration of HJG after LPS injection.** Data are shown as the mean ± SEM (*n* = 5 or 6). ^*^P < 0.05 using Dunnett’s post-hoc test. Abbreviations: KS: kososan; NKS: nobiletin-rich kososan; HJG: hachimijiogan; LPS: lipopolysaccharide.

To examine whether Kampo formulas affect systemic inflammation, serum levels of inflammatory factors were measured by ELISA. Under LPS-stimulated conditions, serum IL-6 levels were significantly increased in the water-administered SAMP8 mice as compared with the water-administered SAMR1 mice (Figure 7; Dunnett’s post-hoc test, *P* < 0.05), and this increase was significantly blocked by HJG treatment (Dunnett’s post-hoc test, *P* < 0.05). Likewise, the HJG-administered SAMP8 mice showed a significant decrease in serum MCP-1 levels as compared with the water-administered SAMP8 mice (Dunnett’s post-hoc test, *P* < 0.05). However, no significant difference in either IL-6 or MCP-1 levels was found between these groups under saline-injected conditions. These results suggest that HJG suppresses systemic inflammation stimulated by LPS.

## DISCUSSION

In the present study, we demonstrated that aging-induced depressive-like behaviors in SAMP8 mice were significantly prevented by treatment with KS, NKS, and HJG. We also found that KS and NKS exerted anti-neuroinflammatory effects and that HJG exerted systemic anti-inflammatory effects, either of which could result in the behavioral recovery of depression. Furthermore, the suppressive effect of NKS on neuroinflammation is likely to be superior to that of KS, as indicated by hippocampal levels of cytokines and chemokines associated with each treatment. RNA-seq analysis revealed unique and characteristic gene expression patterns in the hippocampus of SAMP8 mice treated with each Kampo formula. These findings highlight that Kampo formulas could be used as preventive therapy for depression induced by aging and could provide effective therapeutic strategies for aging -related emotional disturbances.

Our findings suggest that the antidepressant-like effect of KS in SAMP8 mice was due to mitigation of neuroinflammation that resulted from augmentation of the anti-inflammatory response without affecting the inflammatory response. This effect is consistent with our previous finding that the antidepressant-like effects of KS are associated with attenuation of neuroinflammation in socially defeated mice [39]. Thus, enhancement of anti-inflammatory responses by KS may be a mechanism underlying behavioral recovery. Although there was no difference in the antidepressant-like properties between KS and NKS, the inhibitory effects of NKS on neuroinflammation appeared to be superior to those of KS, particularly under LPS-stimulated conditions, as indicated by Western blotting analyses and by cytokine and chemokine levels in the hippocampus. In addition, local elevation of MCP-1, which is produced by microglia [62] and astrocytes [63] under inflammatory conditions in brain microenvironmental niches, is involved in trafficking and recruitment of peripheral monocytes into the brain and thus leads to exacerbation of neuroinflammation [64, 65]. Thus, it could be assumed that NKS exerts anti-neuroinflammatory effects not only via direct actions against glial cells but also via indirect actions such as the reduced recruitment of peripheral immune cells, including monocytes. It is also presumed that these effects of NKS could be supported by the anti-neuroinflammatory activity of nobiletin [40, 41, 66].

In our study, a comparative analysis of the components of KS and NKS revealed that NKS contained substantially higher levels not only of nobiletin but also of sinensetin and tangeretin, which are also polymethoxyflavones, relative to KS (Supplementary Table 2). Nobiletin reduces MCP-1 (also known as chemokine ligand 2, CCL2) production under IL-1β–stimulated conditions *in vitro* [67]. Sinensetin and tangeretin also exert anti-neuroinflammatory effects *in vitro* [68–70]. These findings appear to corroborate the synergistic anti-neuroinflammatory effect of polymethoxyflavones in NKS, but confirmation of this mechanism requires further investigation.

Unlike KS and NKS, HJG protected against systemic inflammation, rather than neuroinflammation, in response to LPS. Blocking IL-6 signaling in the periphery, but not in the brain, exerts rapid and long-lasting antidepressant-like effects in a mouse model of depression [71]. In a clinical trial, subcutaneous injection of tocilizumab, an IL-6 antagonist, significantly decreased depression and anxiety levels in patients with rheumatoid arthritis [72]. Given these findings, our results suggest that systemic anti-inflammatory activity underlies the antidepressant-like effect of HJG.

We note that serum levels of CCL2/MCP-1 were reported to be lower in patients with major depressive disorder than in healthy controls [73]. This finding is seemingly inconsistent with our results, which showed that HJG prevented the LPS-induced increase in serum MCP-1 levels, although no difference was found between SAMR1 and SAMP8 mice. Although further studies are needed to verify the biological significance of a reduction in serum MCP-1 levels by treatment with HJG, our results suggest that HJG had a suppressive effect on whole-body inflammation under LPS-stimulated conditions, and it may partially underlie the antidepressant-like effect of HJG. Given that NKS was more effective than HJG in its antidepressant-like properties, the effects of inhibiting neuroinflammation may have a greater influence on preventing aging-induced depression than the effects of preventing systemic inflammation, although this requires further verification.

RNA-seq analysis revealed different patterns of gene expression in the hippocampus that were associated with the effects of administration of different Kampo formulas in SAMP8 mice. These gene expression profiles suggest that Kampo formulas possess distinct mechanisms that underlie their effects on behavioral recovery. We expected that functional analysis using GO and KEGG of the recovered genes (Supplementary Figure 6) would reveal specific functions for each Kampo formula; however, because of the small number of recovered genes associated with KS treatment, it was not possible to define clear differences in enriched terms among Kampo formulas. In contrast, HJG treatment resulted in the highest number of recovered genes and thus yielded the most enriched terms. However, different terms were obtained in the V-shaped and reverse V-shaped recovery, and in the physiological state and LPS-treated state, indicating the broad complexity of the influence of this Kampo formula. Under the LPS condition, genes that were recovered by these three Kampo formulas were significantly enriched in the following GO terms: “immune system process,” “innate immune response,” and “inflammatory response.” The expression levels of these genes varied both positively and negatively in SAMP8 mice (Figure 4C). However, these fluctuations in SAMP8 mice were restored by Kampo formulas to approach those in SAMR1 mice, suggesting that Kampo formulas regulate the imbalance of the inflammation system that is associated with aging.

Interestingly, about one-third of the genes, particularly those with V-shaped recovery, were unannotated genes (Supplementary Figure 5). Some of the unannotated genes were significantly positively or negatively correlated with immobility in the TST (Supplementary Table 3). It is likely that among these unannotated genes are some with therapeutic potential associated with the pathogenesis of aging-associated depression, which highlights the need for further gene verification. In addition, significant alterations in epigenetic-related genes were found in the hippocampus of SAMP8 mice relative to SAMR1 mice (Supplementary Figure 8). Moreover, the expression of some genes (*Hdac1* and *Dnmt3a*) was recovered by HJG treatment, which may lead to greater dynamics of hippocampal genes related to the inflammatory responses in HJG-administered SAMP8 mice. It is possible that some unannotated genes underwent significant recovery in response to treatment with Kampo formulas may be involved in these observations. Further studies are required to verify this possibility.

More recently, it was demonstrated by positron emission tomography that accumulation of tau, rather than that of amyloid-β, in the brain may underlie the manifestation of major depressive disorder (MDD) in older patients [74]. Importantly, no cognitive impairment was found in patients with MDD with increased tau deposition. Given that depression is a risk factor for neurodegenerative diseases, including cognitive dysfunction and dementia [75, 76], it is plausible that depression with tau accumulation precedes the emergence of cognitive impairment. In our study, 19-week-old SAMP8 mice showed tau accumulation in the brain. This level was reduced, in conjunction with behavioral recovery, by KS and NKS treatment. Our findings suggest that reduction in tau accumulation by treatment with KS and NKS may be a possible mechanism underlying the antidepressant-like activities of these Kampo formulas.

In SAMP8 mice at more than 6 months old, nobiletin reduces the phosphorylation of tau, which protects against cognitive dysfunction [77]. This is consistent with our finding that NKS downregulates hippocampal genes involved in tau phosphorylation. However, it is presumed that the effect of nobiletin alone cannot explain the effects of KS and NKS on tau levels. Future studies are required to determine how KS and NKS decrease tau accumulation and how their effects contribute to the antidepressant-like properties.

We also found that some genes relevant to autophagy, phosphorylation, and splicing of tau in the hippocampus were altered by KS, NKS, and HJG treatment in our RNA-seq analysis. Further studies are necessary to clarify why the genes altered by HJG did not result in reduced tau accumulation.

In the present study, LPS injection was used to evaluate neuroinflammatory priming with aging [21, 78–80]. Several findings have been reported that expression of TLR4, which is a well-known innate immune receptor for LPS, is elevated with aging [81, 82]. Given that no differences between SAMP8 and SAMR1 mice aged 19 weeks were observed in the TLR4 expression, it would be presumed that SAMP8 mice aged 19 weeks possess the neuroinflammatory priming rather than intense neuroinflammation along with upregulation of TLR4 expression. The LPS challenge also appears to mimic a state of stress exposure, as assumed by a notable finding showing that social stress induces neuroinflammation via binding of endogenous ligands to TLR4 [83]. Therefore, it is likely that alleviating effects of Kampo formulas on the neuroinflammatory priming in SAMP8 mice may lead to their antidepressant-like activities in the TST, which itself is a stressful behavioral test. Additionally, infections can increase the risk of developing mood disorders, including depression, in humans [84]. Given this finding, the anti-neuroinflammatory effects of Kampo formulas may lead to a decreased risk of post-infection depression. This possibility could be supported by further studies investigating whether Kampo formulas indeed result in behavioral recovery after LPS injection.

There are several limitations to this study. First, we focused on comparing the effects of the three Kampo formulas on aging-related emotional disturbances and neuroinflammation in SAMP8 mice and found that they exerted preventive effects against depression-like behaviors as well as neuroinflammation associated with aging. Some antidepressants have been reported to attenuate neuroinflammation *in vivo* [24, 85] and *in vitro* [86–89], as well as in human studies [25]. Further studies comparing the effects of these Kampo formulas with those of existing antidepressants are necessary for proper evaluation of the efficacy of Kampo medication as a treatment for late-life depression. Second, we did not perform behavioral testing to assess cognitive impairment in 19-week-old SAMP8 mice, because we focused on the effects of Kampo formulas on emotional disturbances before the onset of cognitive dysfunction in these mice. In many cases, SAMP8 mice over 30 weeks of age have been used for evaluating cognitive dysfunction [90–94]. The preventive effects of KS and NKS on tau accumulation in our study may indicate prophylaxis for aging-related cognitive dysfunction, but this requires further verification in aged SAMP8 mice using cognitive tasks. Third, although nobiletin can act as a circadian amplitude enhancer [95, 96], NKS treatment failed to regulate circadian rhythm disruption in SAMP8 mice. A possible explanation for this is that the nobiletin content in NKS was insufficient to reverse circadian rhythm disruption. A second explanation may be that circadian rhythm disruption in SAMP8 mice is genetically and tightly regulated. Additional studies of the effect of NKS on circadian rhythm disruption induced by normal aging are needed to resolve this issue. Fourth, we focused on hippocampal dynamics related to neuroinflammation in this study, but increasing evidence has demonstrated that microglia have brain region–dependent diversity and region-specific sensitivities to aging [97–100]. Therefore, further comparative studies examining the microglial function specific to emotion-related brain regions (e.g., the hippocampus, amygdala, and prefrontal cortex) in SAMP8 mice could yield a better understanding of the mechanisms underlying aging-related emotional disturbances as well as the active sites of each Kampo formula. Fifth, considerable differences in the findings of western blotting and RNA-seq analyses were found in this study. Although this may be related to a temporal gap in the expression of proteins or genes, the effects of unannotated genes detected in this study cannot be disregarded. Another possibility is that there may be some differences in the period for emergence of behavioral recovery between Kampo formulas, which could involve a lag in the expression of genes and proteins. Further studies are required to investigate this possibility. Last, we used SAMP8 mice to examine the effects of Kampo formulas, because these mice show emotional disturbances at relatively young age [101]. However, future studies would be needed to verify the beneficial effects of Kampo formulas in the wild-type aged mice.

## CONCLUSIONS

Aging predisposes individuals to neuroinflammation and emotional disorders, negatively impacting health and reducing the quality of life in older individuals. Given that depression serves as a risk factor for cognitive impairment in late life, preventive therapy would be a more effective strategy for prophylaxis of cognitive decline and other neurodegenerative diseases. Our findings implicate that each Kampo formula has a characteristic efficacy in terms of some different modes of action for neuroinflammation and systemic inflammation and may provide promising options for preventing late-life depression in humans by using Kampo medication. We anticipate further elucidation of the therapeutic role of these Kampo formulas for emotional disorders associated with aging. Since little has been also reported on the comparative study of some Kampo formulas regarding aging-related emotional disturbances, the unique effects of Kampo formulas would be scientifically and clinically informative for therapeutic strategies of aging-related emotional disorders. In addition, further evidence of NKS, which is not clinically used yet in Japan, would be helpful for NKS being available for aging-related depression in humans in the future.

## MATERIALS AND METHODS

### Animals

Male SAMP8 and SAMR1 mice were obtained at 5 weeks of age from Japan SLC (Hamamatsu, Japan) and were allowed to acclimate for 1 week after arrival. Mice were individually housed for avoiding fights and their concomitant injuries at a constant temperature (23 ± 2°C) and humidity (55 ± 10%) and with a 12-h light/dark cycle (lights on at 08:00) with access to food (LabDiet5058, Lab Supply, Fort Worth, TX, USA) and water *ad libitum*.

All animal experiments were approved by the Institutional Animal Care and Use Committee of Kitasato University and were performed in accordance with the Guidelines for the Care and Use of Laboratory Animals of Kitasato University and the National Research Council Guide for the Care and Use of Laboratory Animals in Japan. Every effort was made to minimize the number of animals used and their suffering.

### Preparation of Kampo extract

The component herbs of KS, NKS, and HJG are shown in Table 1. Each Kampo formula was decocted with 600 ml of distilled water until the volume was reduced by half. The water extract was immediately filtered with a commercial tea strainer and centrifuged at 1000 × *g* for 10 min at 4°C, and the supernatant was lyophilized. The total yield of KS, NKS, and HJG extracts was approximately 28%, 25%, and 19.6%, respectively, relative to the dry weight of the herbal mixture. The preparation of KS and NKS extracts and their component analysis were performed by Kotaro Pharmaceutical Co., Ltd (Osaka, Japan).

**Table 1:** Component herbs and daily quantities for kososan (KS), nobiletin-rich kososan (NKS), and hachimijiogan (HJG).

**Herb**	**KS**	**NKS**	**HJG**
a. Cyperus rhizome	4 g	4 g	
b. Perilla herb	2 g	2 g	
c. *Citrus unshiu* peel	3 g		
d. *Citrus reticulata* peel		3 g	
e. Glycyrrhiza	2 g	2 g	
f. Ginger	0.5 g	0.5 g	
g. Rehmannia root			6 g
h. Alisma rhizome			3 g
i. Poria sclerotium			3 g
j. Dioscorea rhizome			3 g
k. Cornus fruit			3 g
l. Moutan bark			3 g
m. Cinnamon bark			1 g
n. Aconite root			1 g
Total herbal weight per day	11.5 g	11.5 g	23 g

Herbs (a–c, f) were purchased from Tsumura and Co. (Tokyo, Japan). Herb (d) was purchased from Kotaro Pharmaceutical Co., Ltd. (Osaka, Japan). Herbs (e, g, h, n) were purchased from Uchida Wakan-yaku Co., Ltd. (Tokyo, Japan). Herbs (i, j, k, l, m) were purchased from Tochimoto Tenkaido Co., Ltd. (Tokyo, Japan).

### Drug treatment and measurement of body weight

**Figure 8:**
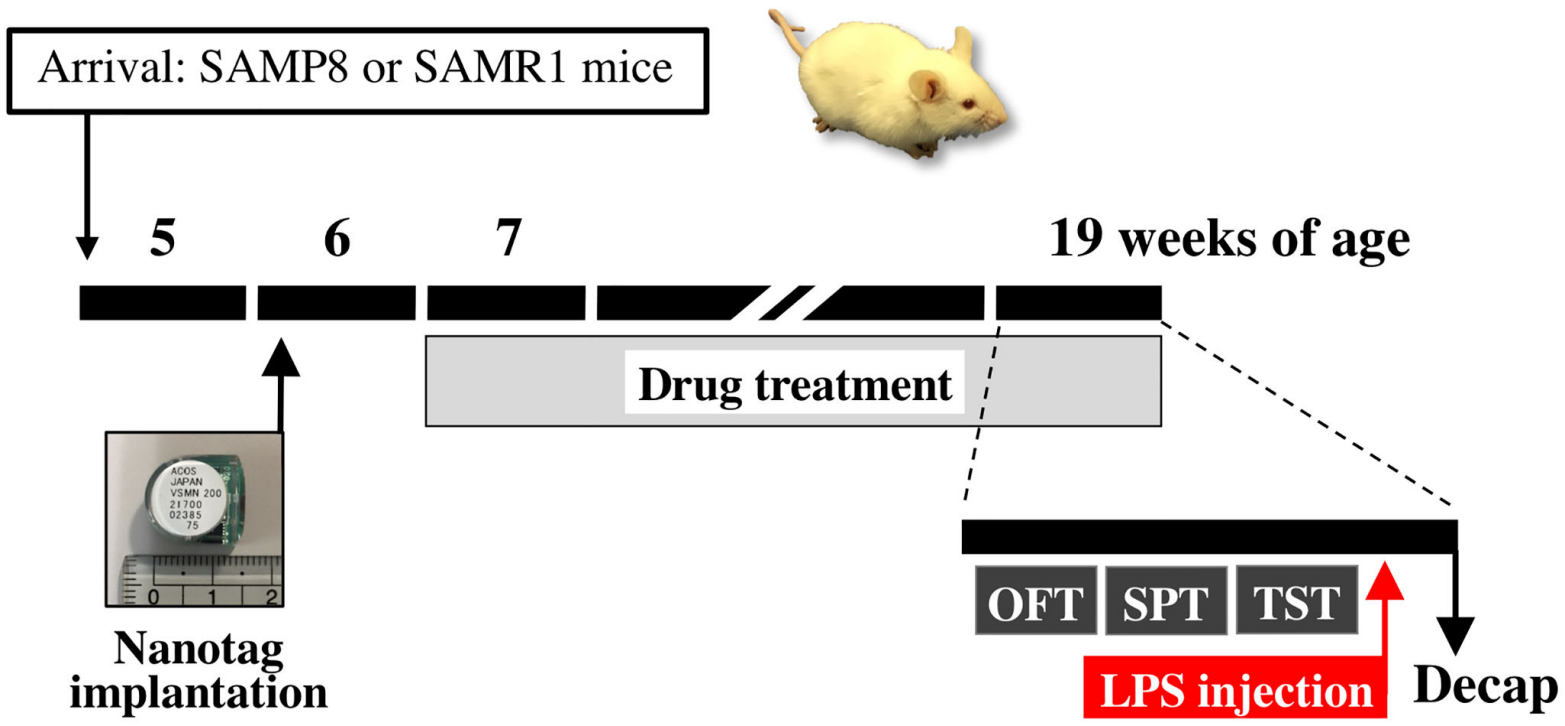
**Schematic representation of the experimental schedule.** Abbreviations: OFT: open field test; SPT: sucrose preference test; TST: tail suspension test; Decap: decapitation; LPS: lipopolysaccharide.

Each extract of the Kampo formula was dissolved in distilled water. The extract (1.0 g/kg body weight) or distilled water was administered once daily by oral gavage to mice from 7 weeks of age for 13 weeks (Figure 8). The dose of KS extract in this study was selected based on our previous studies, which showed that KS extract has an antidepressant-like effect in mouse models of stress-induced depression [35–37, 39, 102]. Other Kampo extracts (NKS and HJG) were set to the same dose as KS for comparison. The dose of each Kampo formula used in mice was equivalent to approximately 15-fold of that in humans. Body weight was measured in mice once a week before the drug treatment.

### Monitoring of locomotor activity

Locomotor activity of mice in their home cages was simultaneously recorded for 24 h using a Nanotag device (Kissei Comtec Co., Ltd., Nagano, Japan), an implantable three-axis accelerometer (weight, 2.6 g). The nanotag was intraperitoneally implanted in half of mice per group aged 6 weeks under inhalation anesthesia with isoflurane (Figure 8). After recovery for 1 week, the locomotor activity of mice at 7, 9, 11, 13, 15, and 19 weeks of age was recorded in 4-min bins over 24 h, and the data were analyzed using the Nanotag viewer program (Kissei Comtec Co., Ltd.).

### Open field test

For the open field test (OFT), individual mice were introduced into an opaque gray open field box (40 × 40 × 40 cm) and were allowed to explore freely for 5 min under high light conditions (120–130 lux). Time spent in three zones (center, intermediate, and peripheral zones) and the total distance traveled were recorded using a video tracking system (EthoVision 3.0; Noldus, Wageningen, Netherlands). Anxiety-like behaviors were evaluated based on the time spent in the three zones. The OFT was performed between 12:00 and 17:00 after acclimation to a habituation room for 1 h.

### Sucrose preference test

The sucrose preference test (SPT) was performed to evaluate anhedonia, a depression-like behavior, as described [21, 103] with some modifications. Briefly, mice were trained to adapt to the presentation of two bottles of water in their home cages for 2 days. After adaptation, each mouse had free access to two bottles, one containing a 2% sucrose solution and the other containing water, with a counterbalance in its home cage from 17:00 to 9:00 over 2 days. The sucrose preference ratio was calculated as follows: (2% sucrose solution intake [ml]/total fluid intake [ml]) × 100, and the average of the ratio per mouse over the 2 days of the experiment was used for analysis.

### Tail suspension test

The tail suspension test (TST) was performed to evaluate apathy, which is a depression-like behavior, as described [21, 104] with some modifications. Briefly, for 6 min the mice were suspended by their tails 50 cm above the floor using experimental clips (Yamashitagiken, Tokushima, Japan) connected to a hook that was connected to a steel bar. A mouse was considered immobile only when it ceased struggling and hung motionless. All behaviors were videotaped, and the duration of immobility during the last 4 min of the TST was determined. The TST was conducted between 12:00 and 14:00 after acclimation to a habituation room for 1 h.

### Lipopolysaccharide injection

After the TST, mice were injected *i.p.* with lipopolysaccharide (LPS, serotype O55:B5, 0.33 mg/kg body weight; Sigma, St. Louis, MO, USA) or saline. Intraperitoneal LPS injection was performed at a volume of 10 ml/kg body weight.

### Brain and serum collection

Twenty-four hours after LPS or saline injection, the mice were decapitated, and their brains and trunk blood samples were collected. The brains were frozen in liquid nitrogen. Two hours after blood sampling, trunk blood was centrifuged twice at 3,000 × *g* for 1 min at 4°C to collect serum. Brain and serum samples were stored at −80°C until further processing.

### Western blotting

Frozen brains were sliced into ~1-mm-thick coronal sections (bregma −1.5 to −2.5 mm), and hippocampi were dissected on dry ice. Proteins were extracted from the hippocampi by sonication of the tissue samples in lysis buffer (CelLytic MT, Sigma) containing 1% (v/v) protease inhibitor cocktail (Sigma) on ice, followed by centrifugation at 18,000 × *g* for 10 min at 4°C and collection of the resulting supernatants. Total protein content was determined using a BCA protein assay kit (Pierce, Rockford, IL, USA). Proteins (10 μg) were separated on 4–15% SDS-PAGE gels (Bio-Rad, Tokyo, Japan) and were transferred to polyvinylidene difluoride membranes (Bio-Rad) using a Trans-Blot Turbo™ Transfer System (Bio-Rad). After being blocked with Blocking One solution (Nacalai Tesque, Kyoto, Japan) for 30 min at room temperature (RT), the membranes were incubated with the following primary antibodies at RT for 1 h or 4°C overnight, as indicated: rabbit anti-nod-like receptor family, pyrin domain-containing (NLRP)3 (1:1000, overnight; Cell Signaling Technology, Tokyo, Japan), rabbit anti-arginase (Arg)1 (1:3000, overnight; GeneTex, Irvine, CA, USA), rat anti-tau (1:2000, overnight; Fujifilm Wako Pure Chemical Corporation, Osaka, Japan), or horseradish peroxidase (HRP)-conjugated mouse anti-GAPDH (1:10000, 1 h; Proteintech, Rosemont, IL, USA). The membranes were then rinsed in Tris-buffered saline containing 0.05% (v/v) Tween 20 and were incubated with appropriate HRP-linked secondary antibodies (except in the case of anti-GAPDH) for 1 h at RT. The blots were visualized with Clarity Western ECL Substrate or Clarity Max Western ECL Substrate (Bio-Rad) using a ChemiDoc™ Imaging System (ChemiDoc Touch, Bio-Rad). Band intensities were analyzed using ImageLab software (Ver. 6.0, Bio-Rad).

### RNA extraction and RNA-sequencing

For RNA extraction, frozen brains were sliced into ~1-mm-thick coronal sections (bregma −2.5 to −3.5 mm), and the hippocampi were dissected on dry ice. Samples were immersed in RNAlater solution (Thermo Fisher Scientific, Carlsbad, CA, USA) and cut into small pieces (~30 mg each) using scissors. RNA extraction was performed using a Pure Link RNA Mini kit (Invitrogen, Carlsbad, CA, USA). To this end, 300 μL of lysis buffer and 450 μL of TRIzol-LS (Thermo Fisher Scientific) were added to the tissue. The tissue was then homogenized on ice using a BioMasher disposable homogenizer (Nippi, Tokyo, Japan). After the sample was incubated for 10 min at RT, it was centrifuged at 12,000 × *g* for 15 min. The supernatant was treated with DNase and the resultant RNA was purified using spin columns. The quality of the total RNA was assessed using a Qubit 4.0 (Thermo Fisher Scientific) and a 4200TapeStation system (Agilent Technologies, Santa Clara, CA, USA). Each fresh-frozen total RNA sample had a RNA Integrity Number (RIN) > 7, indicating that they were of sufficient quality to prepare sequencing libraries.

Total RNA was used for RNA-sequencing (RNA-seq) analysis. Library preparation and Illumina HiSeq sequencing were conducted by GENEWIZ (South Plainfield, NJ, USA), which generated ~20 million (150-basepair, paired-end) reads per library.

### Quality check and filtering of RNA-seq data and mapping analysis

For quality filtering of the RNA-seq data, cutadapt v.1.16 [105] was used to remove Illumina adapter sequences, followed by removal of the poly-A sequence using fastx_clipper software in the fastx toolkit software package v.0.0.14 (FASTX-Toolkit, http://hannonlab.cshl.edu/fastx_toolkit/index.html). To remove low-quality bases or sequences, we trimmed the sequences using fastq_quality_trimmer software (parameters: -t 20 -l 30 -Q 33) and fastq_quality_filter software (parameters: -q 20 -p 80 -Q 33), both of which were included in the fastx toolkit. During the above processing, any read in which one of the pairs was missing was removed using Trimmomatic v.0.38 [106]. Then, reads containing mouse rRNA, tRNA, or phiX sequences, which are the control sequences from Illumina, were removed using Bowtie 2 v. 2.3.4.1 [107]. We then performed a second round of removal of any unpaired reads using bam2fastq (https://gslweb.discoveryls.com/information/software/bam2fastq).

After completion of these filtering steps, 20 million reads of each of the forward and reverse sequences per sample were mapped to the mouse genome sequence to build GRCm38, using HISAT2 v2.1.0 [108]. The mouse genome sequence was downloaded from Illumina iGenomes (https://support.illumina.com/sequencing/sequencing_software/igenome.html). Multiple-mapped reads were removed using samtools (parameters: samtools view -q 4) [109]. Uniquely mapped reads were counted by gene annotation (Ensembl release 81) using featureCounts v.1.6.2 [110]. The counted values were normalized using the Trimmed mean of M values (TMM) method [111] using the edgeR library [112] in R v.3.5.0 (https://cran.r-project.org/bin/windows/base/old/3.5.0/) and were used for expression analysis. The resultant RNA-seq data were subjected to multidimensional scaling analysis. The original RNA-seq datasets were deposited in the DDBJ Sequence Read Archive under accession numbers DRR310804–DRR310853 which are linked to the BioProject accession number PRJDB015423.

### Analysis of differentially expressed genes

*P*-values for the differentially expressed genes (DEGs) were obtained using the likelihood ratio test in the edgeR (https://cran.r-project.org/bin/windows/base/old/3.5.0/) package. First, we extracted genes whose expression levels were significantly down- or up-regulated (*P* < 0.05) in the water-administered SAMP8 mice (SAMP8/water) as compared with the water-administered SAMR1mice (SAMR1/water). Among the genes whose expression levels were significantly down-regulated (SAMR1/water > SAMP8/water), those showing a pattern of significant up-regulation in Kampo- administered SAMP8 mice (SAMP8/Kampo; KS, NKS, or HJG) as compared with SAMP8/water were referred to as "V-shaped recovered" genes (SAMR1/water > SAMP8/water, and SAMP8/water < SAMP8/Kampo) (Figure 4A, green line). Conversely, among the genes whose expression levels were significantly up-regulated (SAMR1/water < SAMP8/water), those that showed a pattern of significant down-regulation in SAMP8/Kampo as compared with SAMP8/water were referred to as "reverse V-shaped recovered" genes (SAMR1/water < SAMP8/water, and SAMP8/water > SAMP8/Kampo) (Figure 4A, orange line).

### Gene ontology analysis and pathway analysis

The DEGs were mapped to the relevant biological annotation from Gene Ontology (GO; http://www.geneontology.org/) and the Kyoto Encyclopedia of Genes and Genomes (KEGG) database (https://www.genome.jp/kegg/) using the DAVID functional annotation online database v.6.8 (https://david.ncifcrf.gov/). Fisher’s exact test was applied to identify significant GO terms and pathways, and the threshold of significance was defined by the *P*-value and false discovery rate.

### Enzyme-linked immunosorbent assay

Cytokine and chemokine levels in the samples were detected using a commercially available enzyme-linked immunosorbent assay (ELISA) kit for interleukin (IL)-1β and IL-6 (BD OptEIA™ ELISA set, BD Biosciences, San Diego, CA, USA) and for tumor necrosis factor (TNF)-α, IL-4, IL-10, and monocyte chemoattractant protein (MCP)-1 (Mouse DuoSet ELISA, R&D Systems, Minneapolis, MN, USA).

### Statistical analysis

All data are presented as the mean ± SEM and were analyzed using Prism 7 (GraphPad Software, San Diego, CA, USA). We used the paired *t*-test for comparisons between two groups. Data were also analyzed by one-way ANOVA, followed by Bonferroni’s or Dunnett’s post-hoc test for comparisons among multiple groups. Correlation analysis was also performed using Pearson’s correlation coefficient. For gene expression analysis based on RNA-seq, *P-*values were obtained using the likelihood ratio test. In all cases, *P* < 0.05 was considered statistically significant.

## Supplementary Materials

Supplementary Figures

Supplementary Table 1

Supplementary Tables 2 and 3
