# Supplementary material for: Kampo formulas alleviate aging-related emotional disturbances and neuroinflammation in male senescence-accelerated mouse prone 8 mice
Source: Aging (Albany NY). 2022 Jan 3;14(1):109–42. doi: 10.18632/aging.203811 (PMC8791223; doi:10.18632/aging.203811)
Supplement: Supplementary Figures [file aging-14-203811-s001.pdf]

SUPPLEMENTARY FIGURES

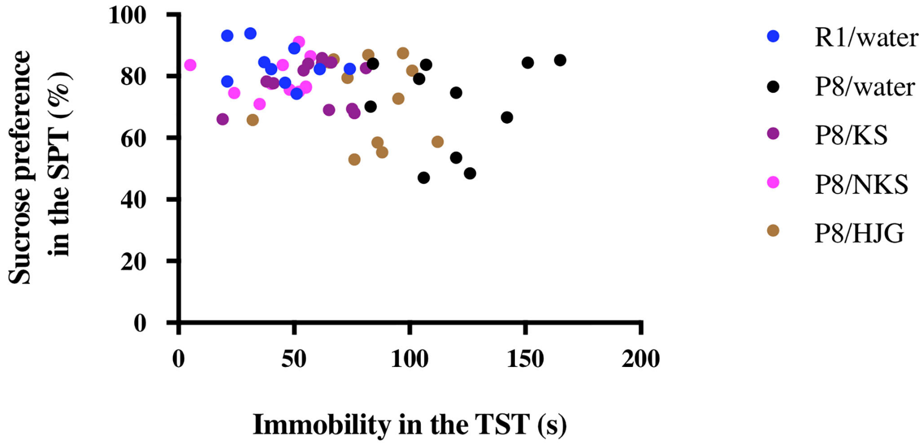

Supplementary Figure 1. Data in the TST were inversely correlated with those in the SPT. A single data point is shown for each mouse. Pearson's correlation coefficient test was performed (r = –0.3048, n = 57, P < 0.05).

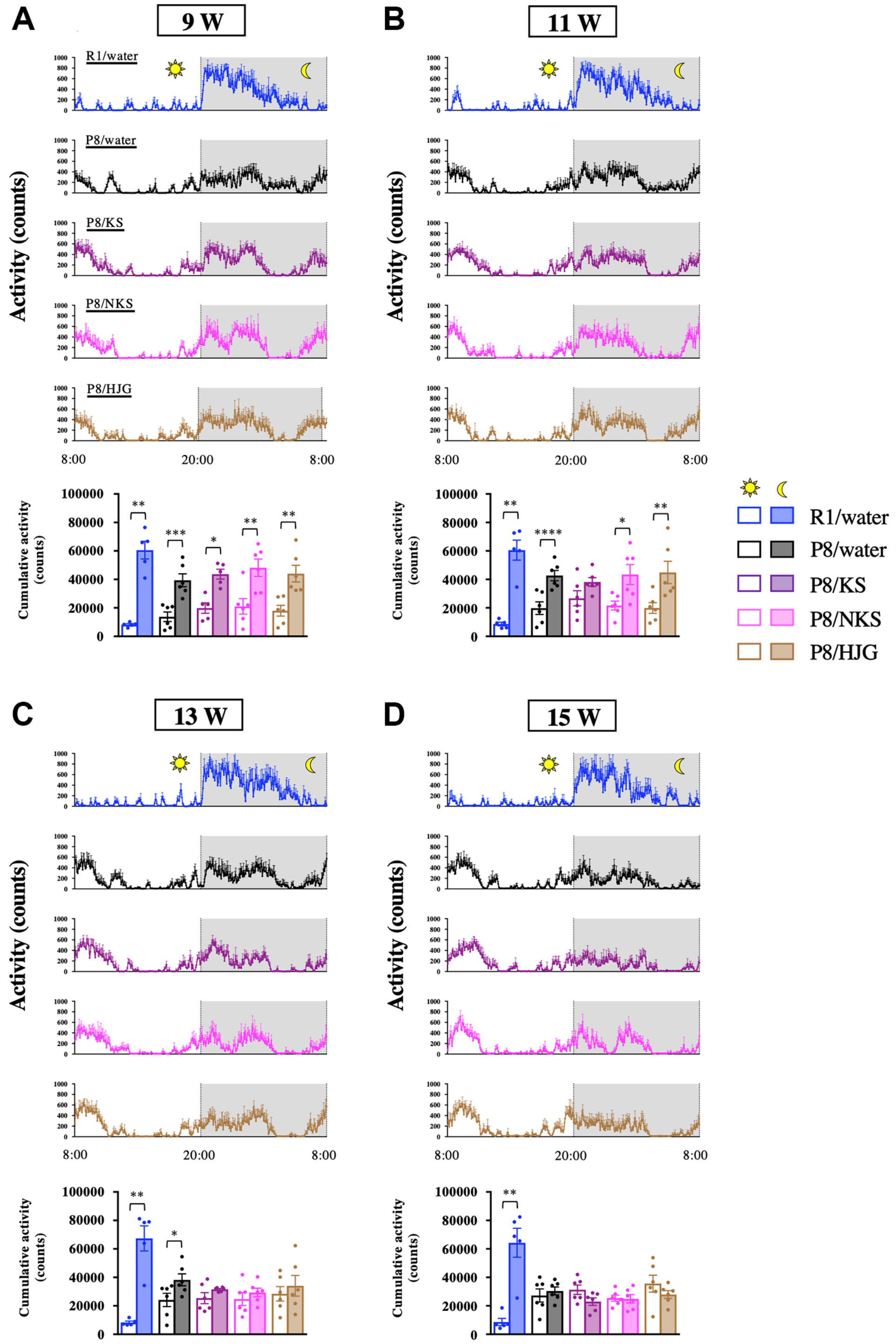

Supplementary Figure 2. The timeline of alterations in circadian rhythm in SAMP8 mice. Time course of locomotor activity of mice in their home cages and the cumulative activity during the diurnal and nocturnal phases are shown at 9 (**A**), 11 (**B**), 13 (**C**), and 15 (**D**) weeks of age. Data are shown as the mean ± SEM (n = 5 or 6). $^{*}P < 0.05$, $^{**}P < 0.01$, $^{***}P < 0.001$, and $^{****}P < 0.0001$, using paired t-test.

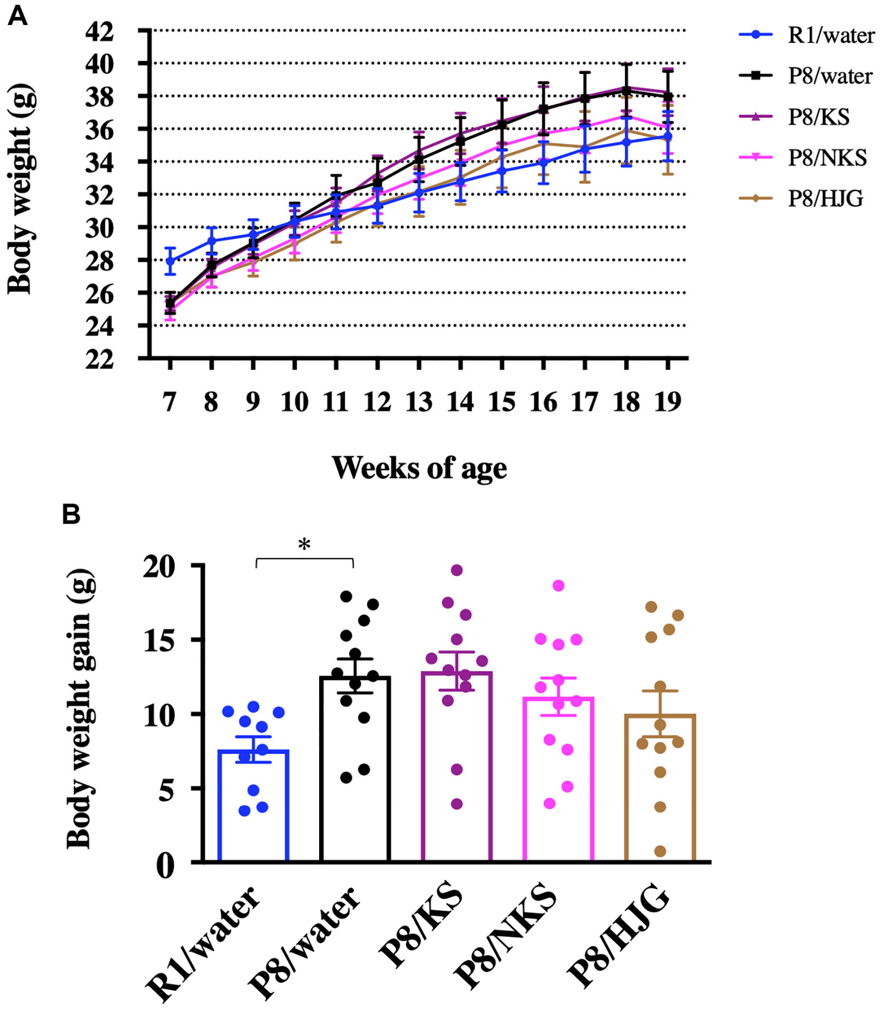

Supplementary Figure 3. Body weight gain is not affected by treatment with Kampo formulas. (**A**) Temporal changes in body weight. (**B**) Body weight gain of mice from 7 to 19 weeks of age. Data are shown as the mean ± SEM (n = 10–12). $^{*}P < 0.05$ using Bonferroni's post-hoc test.

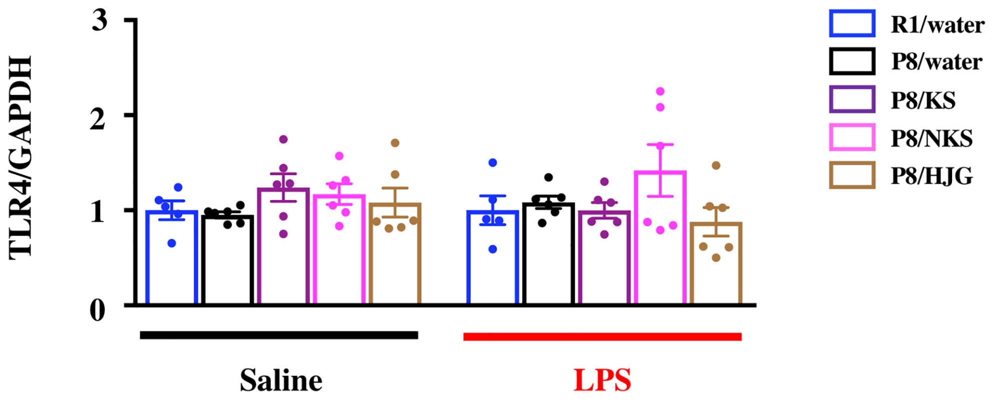

Supplementary Figure 4. TLR4 expression in the hippocampus of SAMP8 mice is not affected by oral administration of Kampo formulas. Expression of TLR4 was normalized based on GAPDH. Data are shown as the mean ± SEM (n = 5 or 6).

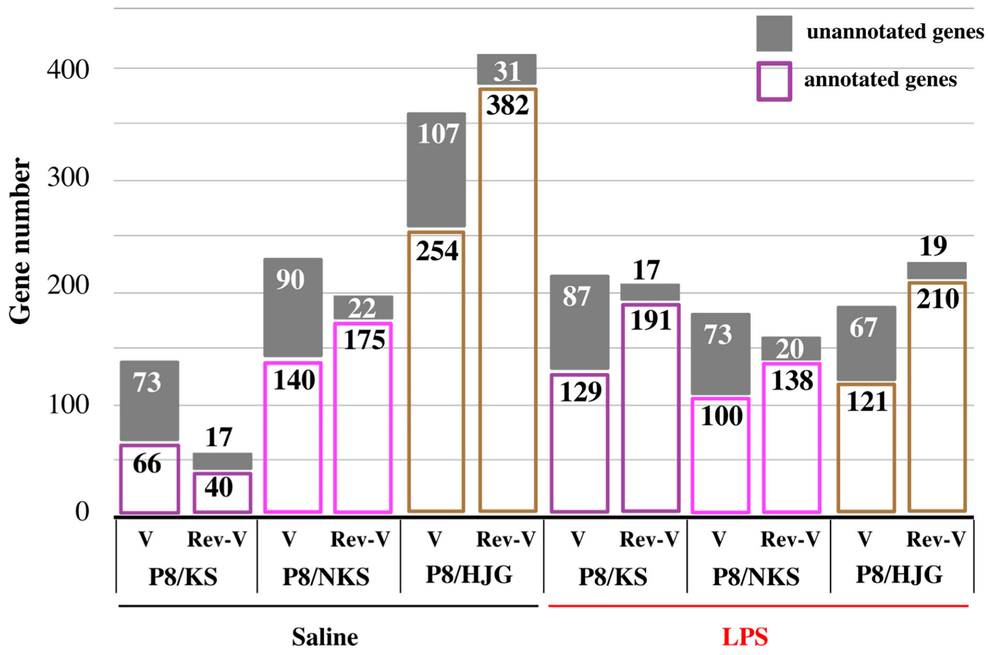

Supplementary Figure 5. Bar plot showing the distribution of the number of annotated and unannotated genes among the significantly recovered genes in the hippocampus of mice treated with Kampo formulas that were then administered saline or LPS. The number in each column represents the number of genes, which corresponds to the data in Figure 5B.

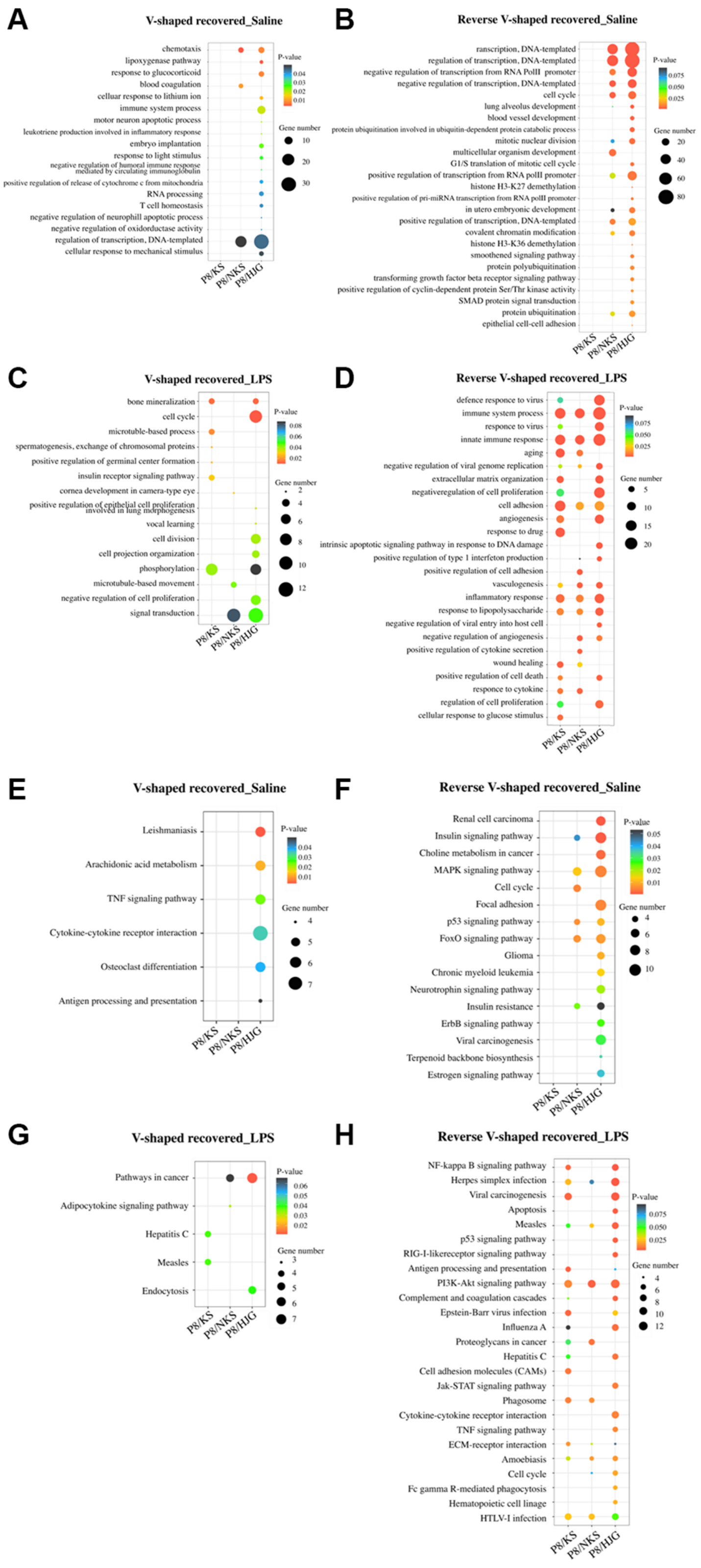

Supplementary Figure 6. Bubble plot showing GO analysis an dKEGG signaling pathway analysis of recovered DEGs in the hippocampus of SAMP8 mice in response to treatment with Kampo formulas. GO analysis of (**A**) all V-shaped recovered DEGs and (**B**) the top 25 reverse V-shaped recovered DEGs from saline-injected mice. GO analysis of (**C**) all V-shaped recovered DEGs and (**D**) the top 25 reverse V-shaped recovered DEGs from LPS- injected mice. KEGG signaling pathway enrichment analysis of (**E**) all V-shaped recovered and (**F**) all reverse (V-shaped recovered DEGs with saline injection. KEGG signaling pathway enrichment analysis of (**G**) all V-shaped recovered DEGs and (**H**) the top 25 reverse V-shaped recovered DEGs with LPS injection.

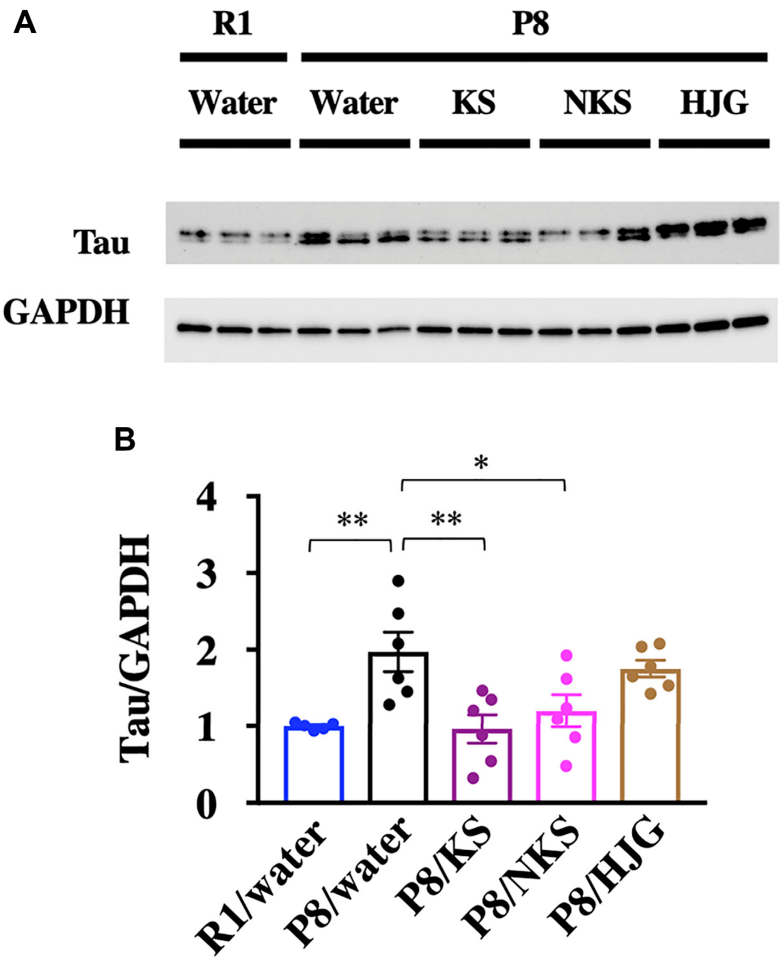

Supplementary Figure 7. Oral administration of KS and NKS, but not HJG, prevents the increase in tau in the brains of SAMP8 mice. (**A**) Representative western blotting images of tau and GAPDH expression. (**B**) Expression of tau was normalized based on GAPDH expression. Data are shown as the mean ± SEM (*n* = 5 or 6). $^{*}P < 0.05$ and $^{**}P < 0.01$ using Bonferroni's post-hoc test.

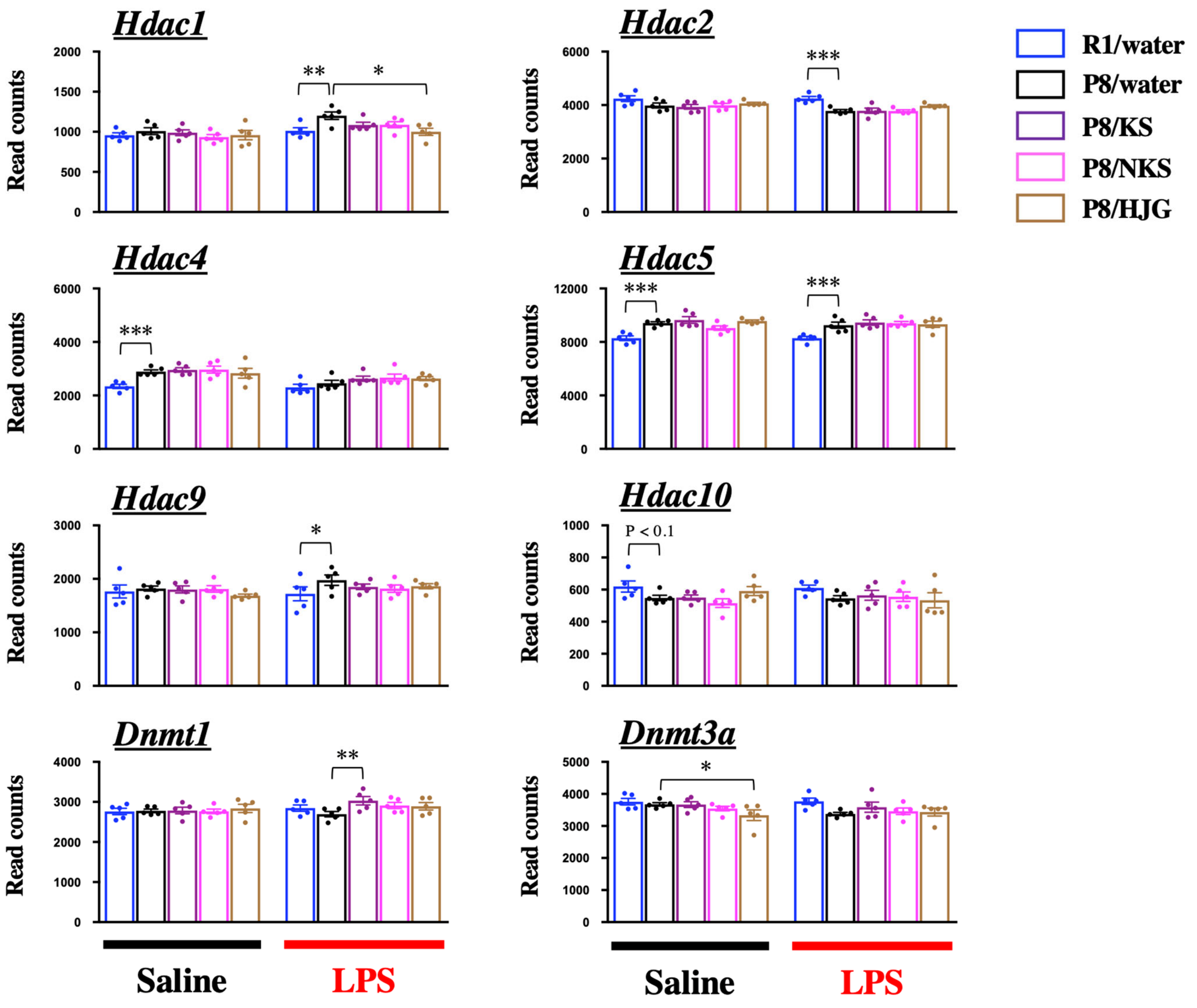

Supplementary Figure 8. Hippocampal gene expression of epigenetic-related genes in Kampo formula–administered mice after LPS or saline injection. Data are shown as the mean ± SEM (n = 5). $^{*}P < 0.05$, $^{**}P < 0.01$, and $^{***}P < 0.001$ using Bonferroni's post-hoc test.
