# Supplementary material for: Kampo formulas alleviate aging-related emotional disturbances and neuroinflammation in male senescence-accelerated mouse prone 8 mice
Source: Aging (Albany NY). 2022 Jan 3;14(1):109–42. doi: 10.18632/aging.203811 (PMC8791223; doi:10.18632/aging.203811)
Supplement: Supplementary Tables 2 and 3 [file aging-14-203811-s003.pdf]

SUPPLEMENTARY TABLES

Supplementary Table 2. Amount of the three polymethoxyflavonces—nobiletin, sinensetin, and tangeretin—in 1-gram water extracts of kososan (KS) and nobiletin-rich kososan (NKS).

	Nobiletin	Sinensetin	Tangeretin
KS	0.09 mg	N.D.	0.02 mg
NKS	2.44 mg	0.142 mg	0.516 mg

For this analysis, water extracts of KS and NKS was analyzed by high performance liquid chromatography under the following conditions: For determination of nobiletin, sinensetin, and tangeretin contents, an ODS column (Mightysil RP-18 GP S, 4.6 × 150 mm, 5 µm; Kanto Chemical Co., INC., Japan), equipped with a detector (an ultraviolet absorption photometer, wavelength: 338 nm) and operated at a column temperature of 40°C, mobile phase of 40% acetonitrile in H_2O, and a flow rate of 0.8 ml/min was used. An injection volume of 10 µL was used for all standard solutions and sample solutions tested. Abbreviation: N.D.: not detected.

Supplementary Table 3. Unannotated genes that showed significant recovery after Kampo formula treatment and a significant positive or negative correlation with immobility in the TST.

Gene_ID	Gene_name	Average read count (*n* = 5)					*P*-value (vs. P8/water)				Correlation (vs. TST)	
		R1/water	P8/water	P8/KS	P8/NKS	P8/HJG	R1/water	P8/KS	P8/NKS	P8/HJG	r	*P*-value
**Saline/V-shaped recovered genes**												
ENSMUSG00000082964	Rpl13-ps5	3.21	0.20	4.01	4.45	1.20	0.002	0.001	0.000	0.086	−0.419	0.0369
ENSMUSG00000073752	Gm10570	24.84	9.82	18.44	18.56	13.34	0.001	0.032	0.030	0.310	−0.592	0.0018
ENSMUSG00000077637	Gm22771	138.37	105.13	122.84	131.52	117.13	0.010	0.149	0.037	0.320	−0.410	0.0419
ENSMUSG00000081431	Gm15483	22.83	13.29	21.87	22.55	21.38	0.040	0.060	0.045	0.073	−0.581	0.0023
ENSMUSG00000102863	Gm37639	12.02	3.22	7.22	9.87	5.47	0.002	0.060	0.008	0.231	−0.590	0.0019
ENSMUSG00000052865	Gm13619	7.21	2.01	6.63	8.08	3.84	0.018	0.028	0.009	0.250	−0.577	0.0025
ENSMUSG00000097430	Gm10544	22.64	7.84	16.04	17.74	8.49	0.000	0.006	0.001	0.779	−0.441	0.0274
ENSMUSG00000097648	9330185C12Rik	9.61	3.03	5.21	10.27	4.65	0.020	0.289	0.014	0.405	−0.419	0.0371
ENSMUSG00000100455	Gm29170	398.86	334.44	380.46	394.50	364.66	0.013	0.070	0.020	0.226	−0.612	0.0012
ENSMUSG00000106565	Gm43582	14.83	4.23	7.02	12.71	6.46	0.001	0.195	0.003	0.284	−0.455	0.0223
ENSMUSG00000103283	ENSMUSG00000103283	87.70	68.78	82.17	91.76	75.62	0.042	0.139	0.016	0.434	−0.503	0.0103
ENSMUSG00000056418	BC043934	33.25	16.43	27.45	27.86	14.98	0.005	0.045	0.039	0.731	−0.415	0.0392
**Saline/reverse V-shaped recovered genes**												
ENSMUSG00000105003	Gm40055	3.21	9.64	6.02	3.63	6.88	0.019	0.281	0.034	0.436	0.552	0.0043
ENSMUSG00000083914	Rps18-ps1	0.40	14.04	9.22	6.47	12.08	0.000	0.139	0.009	0.585	0.525	0.0071
ENSMUSG00000047509	Gm6776	7.62	16.33	9.83	6.86	12.09	0.036	0.154	0.018	0.390	0.476	0.0162
**LPS/V-shaped recovered genes**												
ENSMUSG00000100636	Gm3551	5.55	0.40	3.60	4.79	1.99	0.002	0.013	0.004	0.080	−0.591	0.0019
ENSMUSG00000065694	Gm25411	6.97	1.78	4.61	5.99	3.58	0.011	0.084	0.024	0.215	−0.413	0.0400
ENSMUSG00000097330	Gm26672	17.08	7.91	10.97	15.56	10.93	0.019	0.334	0.041	0.340	−0.477	0.0159
ENSMUSG00000073371	Gm6594	34.74	21.75	33.51	33.68	33.02	0.030	0.046	0.043	0.054	−0.399	0.0482
ENSMUSG00000087063	Gm15857	8.96	2.39	6.79	9.37	6.77	0.003	0.020	0.002	0.020	−0.413	0.0401
ENSMUSG00000098164	Gm5493	9.18	2.97	5.19	9.17	3.59	0.032	0.303	0.032	0.733	−0.530	0.0065
ENSMUSG00000069011	Gm10254	117.32	76.23	99.04	132.70	88.94	0.039	0.211	0.008	0.462	−0.472	0.0171
ENSMUSG00000084828	Gm12367	17.11	9.28	13.17	16.56	11.33	0.030	0.224	0.040	0.494	−0.546	0.0048
ENSMUSG00000102728	Gm37934	3.98	0.00	1.60	2.19	0.99	0.000	0.008	0.003	0.024	−0.465	0.0191
ENSMUSG00000044081	Zfp85os	87.82	48.61	65.45	71.40	59.12	0.000	0.073	0.020	0.240	−0.594	0.0017
ENSMUSG00000102193	Gm7299	5.57	0.40	3.39	5.80	2.98	0.015	0.049	0.014	0.065	−0.565	0.0033
**LPS/reverse V-shaped recovered genes**												
ENSMUSG00000097301	AW121686	54.35	74.35	50.89	45.87	51.94	0.032	0.010	0.001	0.015	0.558	0.0037
ENSMUSG00000085896	5330429C05Rik	7.37	20.00	14.19	7.17	15.70	0.034	0.449	0.030	0.590	0.490	0.0130
ENSMUSG00000077316	n-R5s171	0.20	4.36	3.40	1.20	5.98	0.000	0.651	0.040	0.551	0.590	0.0019
ENSMUSG00000080773	Gm12955	1.19	6.94	2.99	2.00	3.18	0.007	0.153	0.043	0.183	0.554	0.0040
ENSMUSG00000086326	Gm13200	6.18	13.63	9.98	4.99	12.13	0.022	0.342	0.005	0.719	0.651	0.0004

Correlation analysis was performed using Pearson's correlation coefficient.
